# Vertical misfit of laser-sintered and vacuum-cast implant-supported crown copings luted with definitive and temporary luting agents

**DOI:** 10.4317/medoral.17997

**Published:** 2012-02-09

**Authors:** Raquel Castillo-de-Oyagüe, Andrés Sánchez-Turrión, José F. López-Lozano, Alberto Albaladejo, Daniel Torres-Lagares, Javier Montero, Maria J. Suárez-García

**Affiliations:** 1 D.D.S.,Ph.D.Associate Professor. Department of Buccofacial Prostheses, Faculty of Odontology. Complutense University of Madrid (UCM), Madrid, Spain; 2 M.D.,D.D.S., Ph.D.Professor. Department of Buccofacial Prostheses, Faculty of Odontology. Complutense University of Madrid (UCM), Madrid, Spain; 3 M.D.,D.D.S.,Ph.D. Cathedratic Professor. Department of Buccofacial Prostheses, Faculty of Odontology. Complutense University of Madrid (UCM), Madrid, Spain; 4 D.D.S.,Ph.D.Tenured Professor, Department of Surgery, Faculty of Medicine. University of Salamanca (USAL), Salamanca, Spain; 5 D.D.S.,Ph.D.Tenured Professor, Department of Stomatology, Faculty of Odontology. University of Seville (US), Seville, Spain

## Abstract

Objectives. This study aimed to evaluate the vertical discrepancy of implant-supported crown structures constructed with vacuum-casting and Direct Metal Laser Sintering (DMLS) technologies, and luted with different cement types. Study Design. Crown copings were fabricated using: (1) direct metal laser sintered Co-Cr (LS); (2) vacuum-cast Co-Cr (CC); and (3) vacuum-cast Ti (CT). Frameworks were luted onto machined implant abutments under constant seating pressure. Each alloy group was randomly divided into 5 subgroups (n = 10 each) according to the cement system utilized: Subgroup 1 (KC) used resin-modified glass-ionomer Ketac Cem Plus; Subgroup 2 (PF) used Panavia F 2.0 dual-cure resin cement; Subgroup 3 (RXU) used RelyX Unicem 2 Automix self-adhesive dual-cure resin cement; Subgroup 4 (PIC) used acrylic/urethane-based temporary Premier Implant Cement; and Subgroup 5 (DT) used acrylic/urethane-based temporary DentoTemp cement. Vertical misfit was measured by scanning electron microscopy (SEM). Two-way ANOVA and Student-Newman-Keuls tests were run to investigate the effect of alloy/fabrication technique, and cement type on vertical misfit. The statistical significance was set at α = 0.05. Results. The alloy/manufacturing technique and the luting cement affected the vertical discrepancy (p < 0.001). For each cement type, LS samples exhibited the best fit (p < 0.01) whereas CC and CT frames were statistically similar. Within each alloy group, PF and RXU provided comparably greater discrepancies than KC, PIC, and DT, which showed no differences. Conclusions. Laser sintering may be an alternative to vacuum-casting of base metals to obtain passive-fitting implant-supported crown copings. The best marginal adaptation corresponded to laser sintered structures luted with glass-ionomer KC, or temporary PIC or DT cements. The highest discrepancies were recorded for Co-Cr and Ti cast frameworks bonded with PF or RXU resinous agents. All groups were within the clinically acceptable misfit range.

** Key words:**Dental alloy, laser sintering, implant-supported prostheses, vertical discrepancy, vertical misfit.

## Introduction 

“Passive fit”, considered as the intimate and simultaneous contact of the inner surfaces of all retainers with all the abutments, suggests absolute lack of strain development in the absence of an applied external load (1). Marginal discrepancies may lead to plastic distortion of the metal framework, ceramic detachment, fracture of any component of the implant-prosthetic system, plaque accumulation, or inadequate stress dissipation (2). However, the exact level of static stress the implant/bone interface can tolerate is yet to be determined (3).

Base metal alloys are often preferred over noble alloys for conventional and implant-supported fixed dental prostheses (FDPs) because of their higher elastic modulus, hardness, fracture strength, and lower cost (4-6). However, the casting of base metals may be more difficult and unpredictable in terms of accuracy (6-8). Titanium has exceptional biocompatibility, corrosion resistance, and strength-to-density ratio that allow it to obtain lightweight FDPs (9). Its main drawback is the complex casting technique that requires specific investment plaster, a preheated oven, and the achievement of a high melting point in an inert atmosphere (8). Direct Metal Laser Sintering (DMLS) is a promising new technology that may avoid the distortions inherent to casting procedures. DMLS machines employ a high-power laser source, such as a carbon dioxide laser, that fuses small particles of a powder alloy into a mass. Each dental structure is built up in layers from the occlusal surface to the margins by scanning cross-sections from a 3D CAD file that contains the framework’s design generated from the abutments’ digitization (6,7,10,11). Research on DMLS for dental use has focused only on the fit of conventional crowns (6,10) and FDPs (11), and on the bond strength of ceramic to laser-sintered metal cores (7).

Cementation may affect the vertical fit of implant prosthetic structures due to hydrodynamic intracoronal pressures that may prevent the complete seating of restorations (12). Among others, non-eugenol acrylic/urethane-based temporary cements have been proposed for retrievability (13). Nevertheless, they may inadvertently result in accidental displacement (14). Permanent luting materials providing better retention, such as zinc phosphate or glass-ionomer, are being replaced in turn with universal dual-cure methacryloxy-decyl-dihydrogen phosphate (MDP)-based and self-adhesive resin cements due to their improved physicochemical properties (15,16). However, their suitability for implant-supported FDPs deserves further investigation. Given the importance of passive fit for the long-term success of implant prostheses and the lack of precision of current manufacturing techniques and luting procedures (1,8), this paper evaluated the vertical misfit of implant-supported crown copings lutes with different cement types.

The null hypothesis tested affirms that the framework alloy/fabrication technique and the lusting cement do not affect the vertical fit of crown structures fixed onto machined implant abutments.

## Material and Methods

Fabrication of frameworks

Three conic titanium abutments for cemented restorations (height = 6 mm) (ref. PCM7013; Implant Microdent System, Barcelona, Spain) were connected to the corresponding implant replicas (diameter = 3.8 mm) tightening the screws with a torque of 35 Ncm. The abutments were fixed with screws and type IV plaster to a special aluminum platform for preparing 3 series of crown copings using different dental alloys and manufacturing systems. The frames consisted of cement-retained implant-supported structures for mandibular premolar crowns. The chemical composition of the investigated metals is reported in ( Table 1).

**Table 1:**
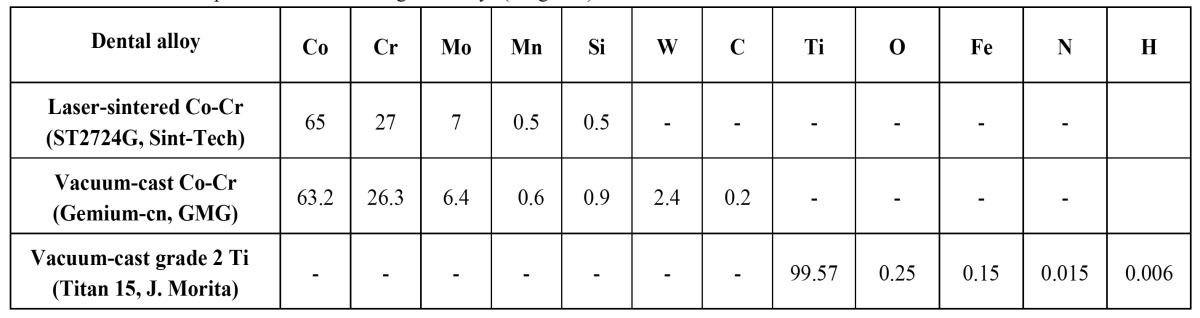
Chemical composition of the investigated alloys (weight %).

Group 1 (LS) was laser sintered using a cobalt-chrome (Co-Cr) powdered base metal alloy for ceramic veneering (batch no 10d0209, ST2724G; Sint-Tech, Clermont-Ferrand, France). The abutments were directly scanned using an optical laser (Cercon Eye; Dentsply, Konstanz, Germany). Implant abutments were placed on the Cercon Eye’s rotating platform, and the scan ran automatically to digitize them. The Cercon Eye’s scanner works on the principle of laser light-sectioning. Two cameras record the route of a laser beam as it is projected onto the surface of the rotating object, while a third camera provides a preview image. The system’s application software (Cercon Art; Dentsply), allows the frameworks to be designed by computer after the abutments are digitized (wall-thickness = 0.8 mm). The structures were obtained in a laser-sintering machine (PM 100 Dental; Phenix SystemsTM, Clermont-Ferrand, France) using the information from the generated file containing the computer-aided design (CAD file). The temperature of the DMLS machine was gradually incremented to 1,650 ºC. The process began by sintering a 20-µm layer of Co-Cr powders onto a stainless-steel platform in an argon atmosphere, and 20-µm increments of metal powders were then sintered from the bottom up until the copings were completed. A 500W Yb-fiber laser was precisely controlled in the X and Y coordinates, allowing for exceptional tolerances to be held (± 0.0254 mm). Once sintered, structures were cooled down to the ambient temperature (decreasing at the rate of 9 ºC per min) inside the furnace.

Group 2 (CC) was vacuum cast using a base metal alloy of white Co-Cr for ceramics (batch no 0711/20cn; Gemium-cn, American GMG Inc., Union City, California, USA). The patterns were waxed-up (Classic modeling wax-blue; Renfert GmbH, Hilzingen, Germany) over burnout casting copings (ref. CCM7011; Implant Microdent System, Barcelona, Spain) and invested in phosphate-based plaster (IPS Press Vest Speed; Ivoclar-Vivadent AG, Schäan, Liechtenstein) using cylinders without a metal ring. Vacuum casting of Co-Cr samples was made in an induction centrifugal casting machine (MIE-200 C/R; Ordenta, Arganda del Rey, Spain) under vacuum pressure (580 mm Hg) at 1,450 ºC. Cast frameworks were then retrieved and cleaned using airborne-particle abrasion with aluminum-oxide powders (50 μm) for 10 s at a working distance of 5 mm and a pressure of 50 ± 3.5 N/cm2 to remove the investment residues.

Group 3 (CT) was made of grade II pure titanium for ceramics (batch no 6-1190-206; Titan 15, J. Morita, Kyoto, Japan). The waxing frames were similar to those of Group 2 (CC), except for the investment material, which consisted on an alumi-na-magnesia-system (Titavest CB; J. Morita, Kyoto, Japan). A pressure-differential machine was used to cast the titanium structures (Cyclarc II; J. Morita, Kyoto, Japan). The casting device was a two-chamber pressure/vacuum machine that smelts the titanium at the temperature of 1,700 ºC with a voltaic arc under an inert argon atmosphere. The “A+C” mode was programmed.

The structures were neither retouched nor polished to avoid external variations.

Lusting procedure

Machined abutments (ref. PCM7013; Implant Microdent System, Barcelona, Spain) were fitted in pairs onto the hexagon-shaped reliefs of a customized clamp support. Frameworks from each alloy group were randomly divided into 5 subgroups (n = 10 each) depending on the cement system employed: Subgroup 1 (KC) used resin-modified glass-ionomer Ketac Cem Plus (batch no. XC9TC; 3M ESPE, St. Paul, MN, USA); Subgroup 2 (PF) used Alloy Primer (batch no. 359AA; Kuraray Medical, Okayama, Japan), plus ED Primer (batch no. 00271A/00144B; Kuraray Medical), plus Panavia F 2.0 dual-cure resin cement (batch no. 00432A-Paste A/00200A-Paste B; Kuraray Medical); Subgroup 3 (RXU) used RelyX Unicem 2 Automix self-adhesive dual-cure resin cement (batch no. 339349; 3M ESPE, Seefeld, Germany); Subgroup 4 (PIC) used acrylic/urethane-based temporary Premier Implant Cement (batch no. 4154CI; Premier Dental Products Co.; Plymouth Meeting, PA, USA); and Subgroup 5 (DT) used acrylic/urethane-based temporary DentoTemp cement (batch no. 4156CI; ITENA, Aulnay-sous-Bois, France). The chemical composition and application mode of the cements tested are detailed in ( Table 2). All of the materials were handled following the manufacturers’ instructions, at a room temperature (RT) of 23.0 °C ± 1.0 °C.

**Table 2:**
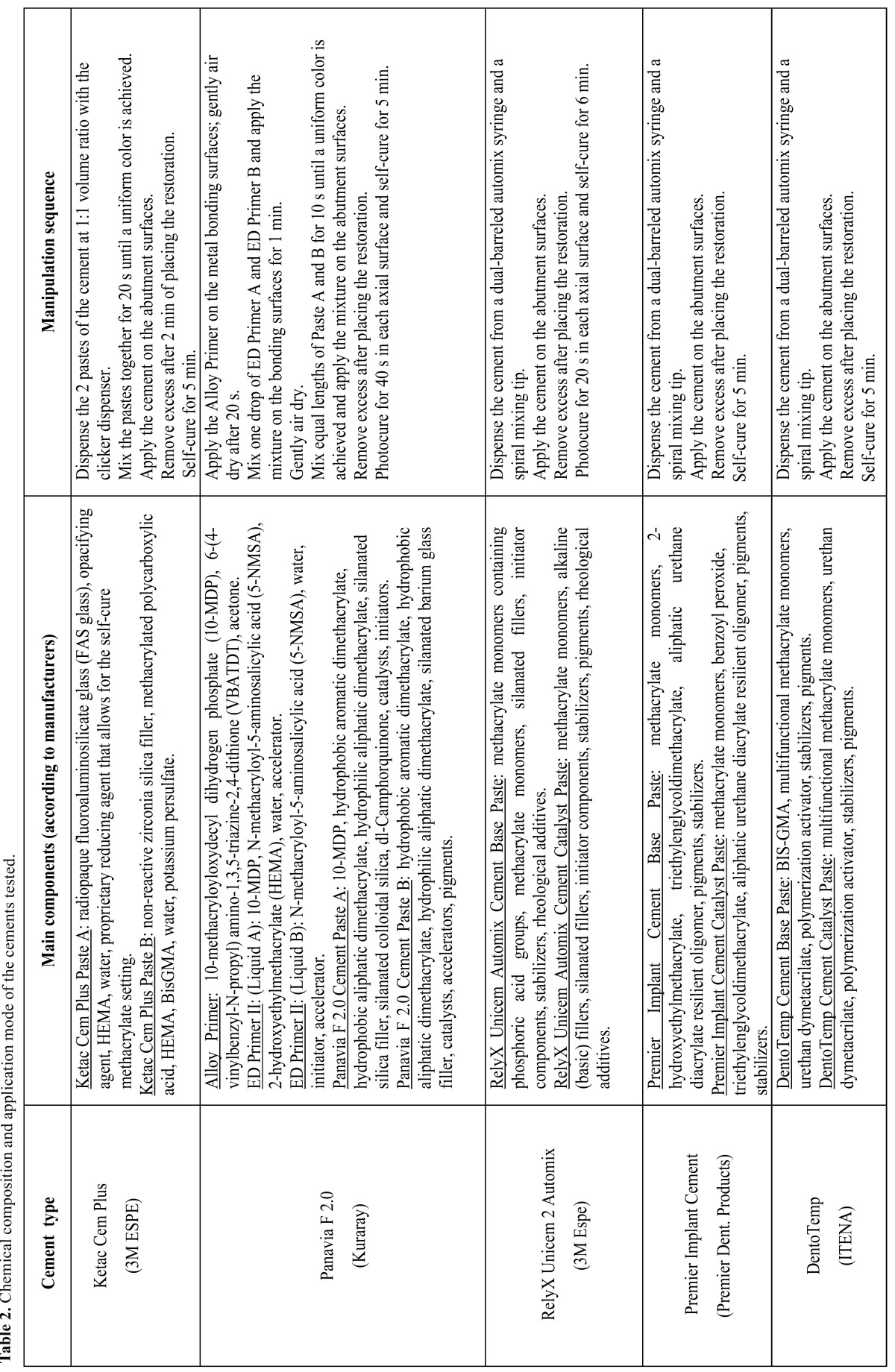
Chemical composition and application mode of the cements tested.

Abutments were varnished with a thin layer of mixed cement before inserting each structure. The clamp press was unscrewed until its base contacted the specimens’ occlusal surfaces. A dynamometric key was then fitted into the upper screw that controlled the press (Defcon I-72000; Impladent, Barcelona, Spain), and a torque of 25 Ncm was kept for 4 min. This contributed to counteract the thyrotrophic behavior of cements in a standard manner by applying uniformly distributed axial load. Following the manufacturers’ instructions, samples of Subgroups 2 (PF) and 3 (RXU) were initially photo-activated around the abutments’ margins (BluePhase; Ivoclar-Vivadent AG, Schäan, Liechtenstein: 600 mmW/cm2) to ensure optimal polymerization. The excess cement was removed with a plastic scaler to avoid scratching or gouging the metal surfaces. All luted specimens were stored in distilled water at 37 °C for 24 h before testing.

Vertical misfit evaluation 

The vertical misfit (or distance parallel to the abutment axis between the lower edge of each structure and the upper margin of the corresponding abutment) was measured with scanning electron microscopy (SEM, JSM-5600LV; Jeol, Tokyo, Japan) Three-dimensional (x/y/z) resolution of 4 nm was obtained at 20 kV of accelerating voltage. The working distance (WD in the z-axis) was 39 mm. Vertical misfit was assessed using different magnifications (from ×100 to ×1000). The cemented samples were mounted on a customized metallic support containing 2 hexagon-shaped reliefs that fitted into the abutment’s hexagons to ensure optimal and repeatable projection angles. Thus, the vertical gap was always perpendicular to the optical axis of the microscopy. To standardize the SEM analysis at the finish line, specific landmarks were traced with an indelible pen in the middle of the axial surfaces of each structure. The marginal opening was measured at the marked points by focussing on the center of the following axial planes: 0º = buccal surfaces; 90º = mesial surfaces; 180º = lingual surfaces; and 270º = distal surfaces. Discrepancies were recorded using image-analysis software (INCA-4.04; Oxford Instruments, Abingdon, UK). To reduce operator bias, vertical misfit was calculated by a specialized technician at 30 points equally distributed by the software on the SEM images taken at each angular position (yielding 120 measurements per structure).

Statistical analysis

A 2-way ANOVA was run to analyze the contributions of framework alloy/fabrication technique, and cement type to the vertical misfit of the crown structures. Multiple post-hoc comparisons were performed using the Student-Newman-Keuls multiple range test. The significance level was set at α = 0.05 for all statistical tests. All data analyses were made with SPSS/PC+ v.17.0 statistical software (SPSS Inc.; Chicago, IL, USA).

## Results

Vertical discrepancy

Means and standard deviations (SD) for vertical discrepancy values are displayed in ( Table 3). Framework alloy/fabrication technique and cement type affected the vertical fit (p < 0.001). Interaction between the two factors was significant (p = 0.017). For each cement type, LS-samples exhibited the best marginal adaptation (p < 0.01), whereas CC- and CT-frames did not differ in vertical misfit. Within each alloy group, PF and RXU provided comparably greater discrepancies (p < 0.05) than KC, PIC, and DT, which showed no significant differences. The vertical misfit of LS structures bonded with either PF or RXU was statistically similar to that of CT samples luted with KC, PIC, or DT.

**Table 3:**
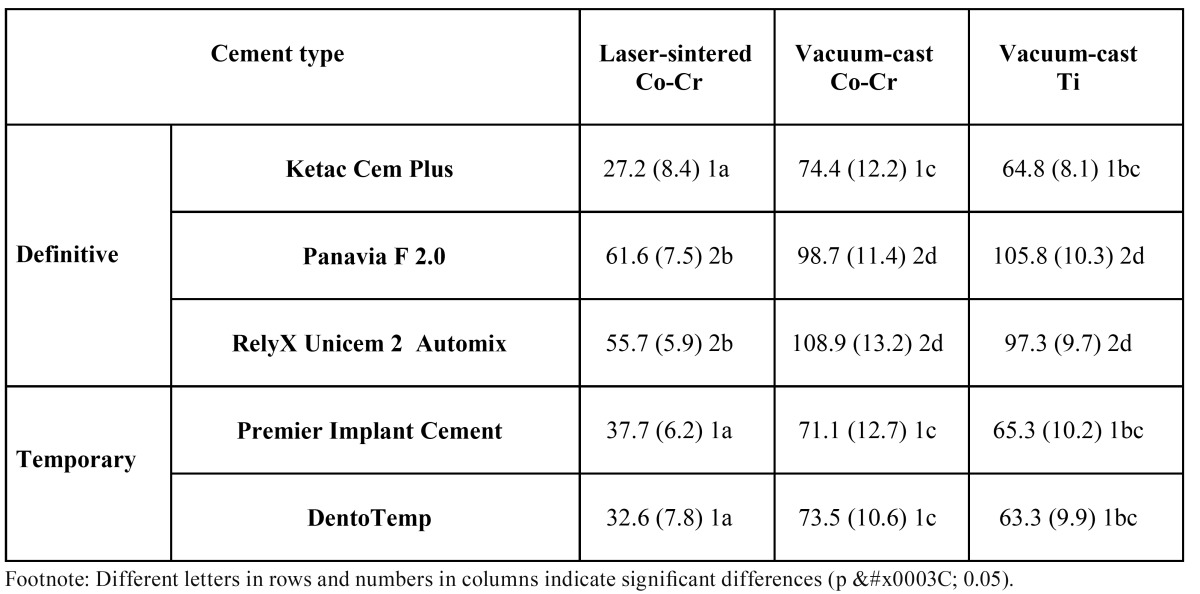
Mean and standard deviation (SD) values of vertical discrepancy (µm) recorded in the experimental groups.

Scanning electron microscopy 

**Figure 1:**
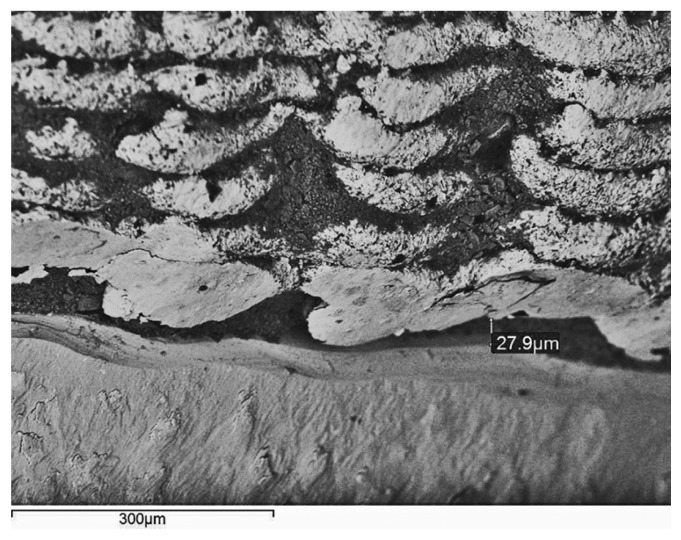
Laser sintered cobalt-chromium structure luted with DentoTemp. There is a vertical discrepancy of 27.9 µm with some washout of the temporary cement (×200; bar 300 µm).

**Figure 2:**
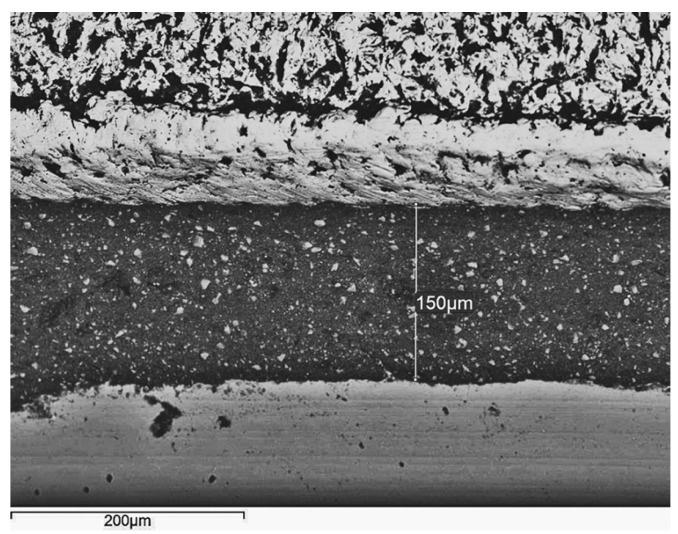
Vacuum-cast cobalt-chromium specimen bonded with RelyX Unicem 2 Automix. A vertical misfit of 150 µm completely filled by the self-adhesive agent is evident (×300; bar 200 µm).

**Figure 3:**
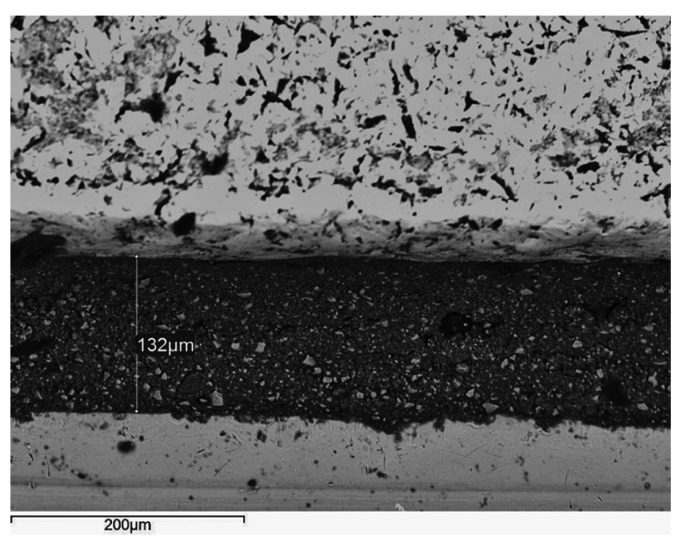
Vacuum-cast titanium coping bonded with Panavia F 2.0. A vertical discrepancy of 132 µm is observable. The resin cement appears almost intact at the marginal area and sparse, scattered porosities are present (×300; bar 200 µm).

LS frames verified the best overall adaptation per cement tested. A perfect fit area was often detectable at the LS structures’ perimeter (Fig. 1). LS specimens exhibited a characteristic rippled texture with microscale undulated surfaces and lobed margins (Fig. 1). CC (Fig. 2) and CT (Fig. 3) structures revealed a comparably rough pattern with irregular contours and edge-shaped microretentions that may be inherent to the casting method. Machined titanium abutments presented smooth margins and flat surfaces (Figs. 1-3).

After 24 h of water storage, KC remained filling the marginal microgaps. PIC and DT (Fig. 1) temporary agents partly dissolved around the margins, and dual-cure resin cements PF (Fig. 3) and RXU (Fig. 2) showed the best marginal seal, displaying protruding filler particles.

## Discussion

This in vitro experiment assessed the vertical fit of cement-retained implant-supported crown structures constructed with vacuum-cast and DMLS technologies. No previous study focuses on the marginal accuracy of laser sintered implant-copings and the comparison among different definitive and temporary cements for providing well-fitted implant-frameworks.

The study’s results support the rejection of the null hypothesis because differences in vertical discrepancy depended on the alloy/manufacturing procedure and the luting agent. CC (Fig. 2) and CT structures (Fig. 3) confirmed the highest discrepancies when either type of cement was used, with no differences ( Table 3). Many factors, such as air bubble entrapment, investing technique, casting pressure, high melting temperature, and potential for oxidation, may affect the quality of Co-Cr cast structures (6-8,17). The complex casting of Ti stems from its low density, higher melting range, and chemical reactivity (18). Inaccuracies of Ti cast pieces may be attributed to pressure differences between fusion and mold chambers, investment material permeability, gas diffusion into the mold chamber, or differences between casting and mold temperatures that accelerate the metal solidification (19). These findings are in accordance to those of Jang et al. (9), who reported comparable clinical fit and detail reproducibility for denture frameworks cast from a Co-Cr alloy and pure Ti. Oyagüe et al. (8) recorded better marginal adaptation for 3-unit cast Ti implant-cemented structures than for their Co-Cr counterparts. In this regard, Jemt et al. (20) stated that the heavier a metal framework, the more distortion is present. For this reason, given its higher density (9), Co-Cr might be expected to provide worse marginal fit than Ti for multiunit constructions.

LS structures resulted in the best marginal adaptation per cement tested (Fig. 1,  Table 3). Because DMLS uses computer-based methods, manufacturers claim that its main advantages are precision, reduction in fabrication steps, ability to prepare up to 90 units in a single operation, simplified post-processing procedures, and improved physicochemical properties (6,7). However, little research exists on the applicability of DMLS to the field of dentistry (6,7,10,11). The PhenixTM Systems approach utilized in this study allows for Co-Cr structures to be prepared by directing the desired proportions of the individual alloy elements into the met pool (7). Ucar et al. (6), who used the same device, reported no differences for the internal gap width of laser-sintered and cast Co-Cr conventional crowns, but vertical misfit was not measured in their investigation.

The misfit ranges of implant-supported FDPs are usually related to screwed prostheses. However, implant-cemented restorations are closer to conventional FDPs concerning the obtaining, setting, and functioning, especially when developing in vitro tests (8). Hence, findings of the present study are consistent with those of Örtorp et al. (11), who found that DMLS provided better marginal fit than casting or even milling Co-Cr for the fabrication of conventional FDPs.

A single operator fabricated all frameworks; wax-patterns were invested immediately to minimize wax contraction (11); and a ring-free technique was selected to reduce distortion (21). Frames were not retouched to avoid external variations that could misrepresent the results (22). A customized tool was used to standardize the pressure exerted during cementation (8,23). Because the cements tested (except RXU) were mixed by hand, minor changes inherent in the mixture and cement thickness may have slightly modified the final fit in an unpredictable manner. Nonetheless, such procedure simulates clinical conditions.

There is no consensus on where the marginal opening should be calculated (12). Published results can only be interpreted relative to the specific measuring method applied (10,11,14,22,24). Holmes et al. (24) defined the “vertical marginal discrepancy” as the vertical distance parallel to the path of draw of the casting, measured at various points along the margins between the casting and the respective abutment. A blind observer consistently examined the vertical discrepancy in equidistant points on each micrograph to avoid bias. Similar measuring methods have been followed for conventional (23) and implant-supported FDPs (8).

After 24 h of water storage, the SEM micrographs showed better marginal integrity of the resin-modified glass-ionomer KC, the MDP-containing PF (Fig. 3), and the self-adhesive RXU (Fig. 2) than for the PIC and DT (Fig. 1) temporary agents. The constant acid dissociation of resin monomers produces phosphate radicals that may interact with the hydroxyl (OH-) groups of the abutments’ surfaces, thus promoting the retention between resin-containing cements and titanium abutments (25). The presence of cement filling the marginal gaps might compensate for misfit at the superstructure/abutment assembly. This may reduce stress forces that could be transmitted to the implant/bone interface as a consequence of marginal cement loss (26). This was not the focus of the study, so further research on the cements’ physical properties and long-term stability is required to support these assumptions.

The resin-modified glass-ionomer and the acrylic/urethane-based provisional cements displayed the best fit while dual-cure resin agents presented comparably greater discrepancies when either type of structure was used ( Table 3). Accordingly, Bottino et al. (27) noticed that glass-ionomer supplied a better cervical adaptation than resin cements when conventional metal crowns where luted onto stainless steel master dies. The lower viscosity and higher plastic deformation in compression of the tested glass-ionomer and provisional cements with respect to the resin agents may explain such results (28). Hence, White et al. (29) recommended applying resin cements swiftly and carefully as they rapidly gain viscosity in the curing process.

The tolerable misfit level that may prevent biological or mechanical failures of implant-supported restorations still remains unknown (3) as there is no longitudinal clinical study that reports implant failure specifically ascribed to framework misfit (1). However, discrepancies of all samples in this study are situated below 150 µm, which is the proposed limit for clinical acceptability of cement-retained implant-supported crowns (30).

Within the limitations of this study, the following conclusions could be drawn: (a) Direct Metal Laser Sintering of Co-Cr may be an alternative to vacuum-casting of base metals to obtain passive-fitting implant-supported crown copings; (b) the best marginal adaptation corresponded to laser sintered structures luted with resin-modified glass-ionomer KC, or temporary PIC or DT cements; (c) the highest vertical discrepancies were recorded for Co-Cr and Ti cast frameworks bonded with PF or RXU resinous agents; and (d) all groups were within the clinically acceptable misfit range.
